# Absence of evidence for post-training tDCS effects on motor memory consolidation and premotor–primary motor cortex interaction: a resting-state EEG study

**DOI:** 10.3389/fnhum.2026.1698460

**Published:** 2026-02-13

**Authors:** Alhuda Dabbagh, Mina Jamshidi Idaji, Elinor Tzvi, Vadim Nikulin, Jost-Julian Rumpf, Joseph Classen

**Affiliations:** 1 Department of Neurology, Leipzig University Medical Center, Leipzig, Germany; 2 Berlin Institute for the Foundations of Learning and Data, Berlin, Germany; 3 Department of Neurology, Max Planck Institute for Human Cognitive and Brain Sciences, Leipzig, Germany

**Keywords:** connectivity, motor consolidation, motor sequence learning, phase slope index, premotor cortex, primary motor cortex, tDCS, transcranial direct current stimulation

## Abstract

**Introduction:**

Motor memory consolidation may be influenced by offline application of non-invasive brain stimulation to the primary motor cortex (M1). One potential underlying mechanism involves changes in oscillatory neuronal activity within the premotor and primary motor cortices, as well as their interaction.

**Methods:**

Twenty-four healthy young participants (age 22.9 ± 2.9 years, mean ± SD) participated in two experimental sessions: a post-training sham and real transcranial direct current stimulation (tDCS) intervention. The anode was placed over the left M1, and stimulation was applied immediately after training of an explicit sequential finger tapping task. The task was repeated 8 h later to assess between-session performance changes, serving as an indicator of the effectiveness of the post-training offline motor memory consolidation process. High-density resting-state electroencephalography was recorded before training and after tDCS to examine beta frequency power, functional connectivity, and directed information flow between M1 and the dorsal premotor cortex (dPMC).

**Results and discussion:**

We observed no meaningful post-training tDCS effects on motor consolidation relative to the sham intervention. Likewise, we found no evidence that post-training tDCS altered beta-band functional connectivity or directed information flow between the left M1 and dPMC. Our findings, therefore, do not provide evidence for a meaningful effect of post-training tDCS of M1 on the offline motor memory consolidation process. However, independent of intervention type, significant post-training increases in beta-frequency power were observed across M1 and dPMC subregions. An exploratory follow-up analysis indicated that stronger directional interactions from M1 to the dPMC could be related to enhanced motor consolidation—a hypothesis that should be further investigated in future studies.

## Introduction

Motor learning is a critical aspect of human behavior, involving the acquisition, refinement, and retention of new motor skills. This process is based on repeated practice and the brain’s ability to adapt and optimize motor memory. Motor sequence learning tasks, where individual elements are integrated into a cohesive motor unit through practice, have provided insights into this process. Evidence suggests that motor sequence learning occurs in distinct phases: an initial fast learning phase during practice (online learning) followed by a subsequent consolidation period in which training-induced motor memory traces are modified in the absence of further practice (i.e., offline), ultimately leading to offline gains, stabilization, or losses (Censor et al., 2012).

Multiple studies have explored non-invasive brain stimulation (NIBS) techniques like transcranial direct current stimulation (tDCS) and repetitive transcranial magnetic stimulation (rTMS) as tools to investigate and modulate online and offline motor learning. tDCS, particularly with anodal stimulation targeting M1, has shown potential in enhancing both online skill acquisition and offline motor memory consolidation (Nitsche et al., 2003; Reis et al., 2009). However, findings have been inconsistent, with several studies failing to replicate these effects or reporting null or even adverse outcomes (Wade and Hammond, 2015; Ambrus et al., 2016; Buch et al., 2017; Kim et al., 2024).

Motor learning involves the coordinated activity of multiple brain regions, including a network that specifically supports offline motor memory consolidation (Muellbacher et al., 2002; Kantak et al., 2012; Debas et al., 2014; Tzvi et al., 2015). We can gain a deeper understanding of coordinated cortical processing—beyond what can be discerned from individual brain regions alone—by examining the neuronal oscillatory connectivity between regions. Electroencephalography (EEG) can capture oscillatory patterns, offering a powerful means to investigate communication between cortical regions. Connectivity measures reflect signal transmission between brain areas that are closely linked to motor performance (Hipp et al., 2012; Guggisberg et al., 2015). Here, we focused on the interaction between the primary motor cortex (M1) and dorsal premotor cortex (dPMC). M1 is traditionally recognized for its role in the execution of voluntary movements. Beyond this, M1 is also involved in motor memory consolidation (Pascual-Leone et al., 1994; Muellbacher et al., 2002; Penhune and Doyon, 2005; Eisenstein et al., 2024). Conversely, the premotor cortex is active in planning and preparing movements, aiding in the organization of motor plans before execution, and also contributes to new motor memory formation (Honda et al., 1998; Meehan et al., 2011). While M1 is responsible for movement execution and refinement, and dPMC for motor planning, their interaction is crucial for effective motor performance. Furthermore, a meta-analysis of motor learning-associated neuroimaging studies concluded that dPMC contributes to motor learning beyond the level of mere movement performance (Hardwick et al., 2013). Therefore, understanding excitability and plasticity in M1, dPMC, and their malleability and dynamic interplay is key to understanding how motor skills are learned and retained over time (Gerschlager et al., 2001; Münchau et al., 2002; Huang et al., 2009; Huang et al., 2018).

The endogenous modulation of brain oscillations, particularly in the beta frequency band (13–30 Hz), has been linked to various stages of motor learning and control (Pogosyan et al., 2009; Engel and Fries, 2010; Joundi et al., 2012). Beta oscillatory activity has been proposed as a candidate marker of plasticity engagement and may relate to consolidation (Ghilardi et al., 2021). Some studies suggest that tDCS can modulate beta oscillatory activity during and after motor tasks (Roy et al., 2014; Song et al., 2014), yet findings are inconsistent and appear to depend on the type of stimulation, the timing relative to motor practice, and inter-individual differences in susceptibility to tDCS (Buch et al., 2017).

A central question is whether post-training neural oscillatory activity within M1 or dPMC, as well as their interregional connectivity, plays a role in the offline consolidation process of motor sequence skills acquired through active training (i.e., online). In addition to regional spectral power analysis, we included EEG measures of functional connectivity. Several methods have been used to explore information transfer between brain regions and obtain insights into functional brain network architecture. Reliable methods should attenuate zero-lag contributions, thereby reducing the impact of common source effects. The imaginary part of coherency (imCoh) has been identified as one of the most sensitive and reliable methods for detecting stimulus-induced functional connections (Nolte et al., 2004; Yoshinaga et al., 2020). As functional connectivity is insensitive to the direction of information flow, we additionally computed the phase slope index (PSI) as a measure of directed information flow (Nolte et al., 2008). Unlike other methods, PSI remains unaffected by non-interacting signals and provides a more accurate assessment of effective connectivity in EEG studies (Haufe et al., 2011).

This study aimed to investigate the effects of post-training tDCS on offline motor memory consolidation and the interaction of oscillatory neural activity between M1 and dPMC.

## Methods

### Participants

We recruited 24 young, healthy participants (mean age ± SD 22.9 ± 2.9 years, range 18–32; 11 male). All participants were right-handed as assessed with the Edinburgh Handedness Inventory (Oldfield, 1971). Exclusion criteria included sleep disorders as well as serious medical, neurological, or psychiatric conditions, specifically depression. Symptoms of depression were screened for using the short version of the Beck Depression Inventory (exclusion cutoff ≥ 13; Beck et al., 1974). Participation was also restricted to exclude professional musicians and trained typists. The level of alertness was assessed before the initial training session and before each delayed retest session using the Stanford Sleepiness Scale (Hoddes et al., 1973). All participants provided written informed consent to participate in the study. The study protocol conformed to the principles of the Declaration of Helsinki and was approved by the Ethics Committee of the Medical Faculty at the University of Leipzig (registration number: 207/17-ek).

### Study design

**Figure 1:**
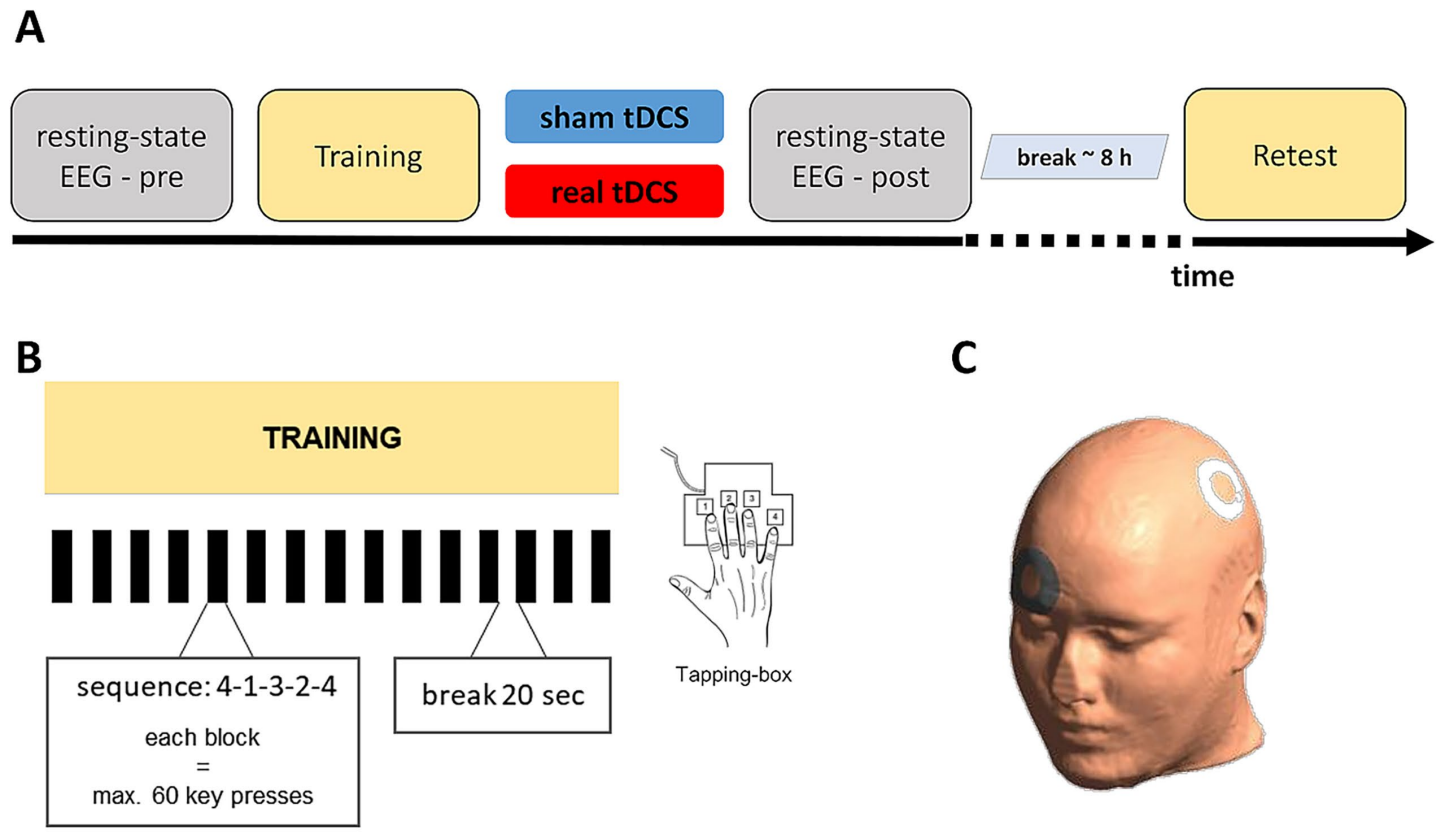
Experimental design. **(A)** Timeline of the study. **(B)** Motor sequence learning task. Participants practiced a 5-element finger-tapping sequence (4-1-3-2-4, 1 = index finger, 2 = middle finger, 3 = ring finger, 4 = little finger; see Tapping-box) with their right hand as fast and accurate as possible. The training session encompassed 14 blocks of task practice, whereas the retest session contained 4 blocks. Each block was terminated after 60 key presses and the next block began after a 20-s rest period. **(C)** Stimulation electrode montage: The anode (white) and cathode (gray) were centered over C3 and Fp2, respectively. EEG, electroencephalography; tDCS, transcranial direct current stimulation; C3, Fp2, EEG electrode position according to 10-20-system.

Each participant took part in two experiments separated by an interval of at least 2 weeks (26.8 ± 14.7 days, mean interval ± SD), corresponding to two different post-training tDCS interventions (i.e., real tDCS and sham tDCS). Each experiment consisted of a motor sequence training session followed by immediate post-training tDCS intervention and a delayed retest session after an interval of 8 hours to evaluate between-session task performance changes, used as an indicator of how effectively the initial training-induced performance gains were consolidated offline across the post-training rest period. (Figure 1A). Resting-state EEG was recorded for 10 min before the initial training session (pre-EEG), and for 10 min after termination of the post-training tDCS intervention (post-EEG). Participants were blinded to the type of tDCS intervention. To assess the effectiveness of blinding for the post-training tDCS intervention, participants were asked after each session whether they believed they had received real or sham stimulation.

### EEG recordings and stimulation electrode preparation

Participants were seated in a comfortable chair in front of a computer screen and were equipped with a 64-channel EEG cap (ANT waveguard™ EEG cap; ANT Neuro, Netherlands). EEG data were recorded with the EEGO™ Mylab amplifier and software (ANT Neuro, Netherlands). The tDCS electrodes (ring rubber electrode, outer diameter = 5 cm, inner diameter = 4.5 cm) were centered around the C3- (anode) and Fp2- (cathode) EEG electrodes (according to the international 10–20 EEG system). The two-ring electrodes were prepared with Ten20 conductive paste (Weaver and Company, Colorado, United States). Impedance of the stimulation electrodes was controlled by a neuroConn DC-stimulator (neuroConn GmbH, Germany) and was kept under 3 kΩ. After preparation of the tDCS electrodes, the EEG cap was placed carefully on the head to avoid movement of the stimulation electrodes. The EEG electrodes were prepared using an EEG electrode gel (Electrogel™ GEL EEG ECI, Electro-Cap Center B. V., Nieuwkoop, Netherlands). Impedances of the EEG electrodes were kept under 10 kΩ. Data were recorded at 512 Hz, and the default online reference was set to Cpz. Furthermore, an electrooculogram (EOG) electrode was placed beneath the left eye to detect eye movements. For offline re-referencing, an average of the left and right mastoid electrodes was computed. Participants were asked to fixate a cross displayed on the computer screen for 10 min, while eyes-open resting EEG was recorded (pre-EEG), and were instructed to think about whatever came to mind and to relax (especially facial and jaw muscles). Throughout the EEG recordings, the experimenter monitored the online EEG signal and indicated when the participant was not relaxed or noted the circumstances leading to artifacts. After completion of the training session and the post-training tDCS intervention, eyes-open resting EEG was recorded for an additional 10 min (post-EEG).

### Motor learning task

Motor sequence learning was assessed by an adapted version of the well-established sequential finger-tapping task introduced by Karni et al. (1995). Participants were instructed to practice a five-element finger-tapping sequence on a customized keyboard with their right hand as rapidly as possible while making as few errors as possible. Before the training session, participants had to demonstrate explicit knowledge of the sequence by repeating it correctly three times in a row on the keyboard. The training session encompassed 14 successive practice blocks separated by 20-s rest periods (Figure 1B). To control for the number of finger movements, each block was terminated after 60 key presses. Therefore, a maximum of 12 correct sequences could be executed within one practice block. During rest blocks, indicated by a red fixation cross on the screen, participants were instructed to fixate on the cross and relax their hand until the next training block began, signaled by the cross turning green. The delayed retest session to assess offline performance changes (i.e., consolidation) consisted of four blocks of the same task.

Participants’ motor performance was analyzed using customized MATLAB (MathWorks®, Natick, United States) scripts. Task performance speed [mean time to complete correct sequences (TCS) in a given block] and accuracy (number of erroneous sequences per block divided by the maximum number of correct sequences per block, i.e., 12) were assessed.

Motor sequence performance was defined as a composite measure (Performance Index [PI]) as applied in previous research (Dan et al., 2015; King et al., 2017) to integrate speed and accuracy of task performance according to the following formula:


$$PI_{x}=e^{-(\mathrm{TC}S_{x})}\times e^{-(\frac{\text{error}s_{x}}{12})}\times 100$$

where x is a given block of task performance.

Repeated measures ANOVAs (rmANOVA) were applied to compare performance between both types of tDCS intervention with the main factors Intervention (sham and real tDCS), Session (training and retest), and Block (training session blocks B1 to B14 or retest session blocks R1 to R4). RmANOVAs were checked for violation of sphericity, and degrees of freedom were corrected (Greenhouse–Geisser) if necessary. Offline between-session changes in task performance were used to index the effectiveness or magnitude of the offline motor memory consolidation process. Offline between-session changes in task performance, abbreviated as ΔPI_offline_, were calculated as the difference between average PI at the end of training session (EoT, mean PI across last two blocks of training session) and the average PI in the first two blocks of the delayed retest (BoR). The data of two participants had to be excluded from the analysis due to technical issues. All statistical analyses were conducted with SPSS 27 (SPSS, Chicago, IL, United States).

### Transcranial direct current stimulation

Post-training real and sham tDCS were delivered for 15 min after completion of the motor training session (DC-Stimulator-Plus, Neuroconn, Germany). The anode was centered over C3, which corresponds to the hand area of M1 (Cui et al., 2000). The cathodal electrode was placed on the supraorbital region ipsilateral (Fp2) to the trained hand (Figure 1C). During stimulation, participants were instructed to relax while watching a series of alternating photographs of landscapes on the computer screen. At the onset of stimulation, the electric stimulation current was increased in a ramp-like fashion over a period of 10 s until it reached 1 mA. The sham stimulation started identically; however, the electric current faded over 10–30 s after the stimulation current of 1 mA was reached. This was done to blind the participants toward real or sham stimulation (Gandiga et al., 2006; Nitsche et al., 2008). The experimenter was not blind to the type of stimulation (sham or real tDCS).

### EEG data analysis

#### Pre-processing

Pre-processing and all subsequent analyses were performed using custom MATLAB (Mathworks®, Natick, MA) scripts and the EEGLAB toolbox (Delorme and Makeig, 2004) after exporting the raw EEG data from the acquisition software. Data were re-referenced to the mastoid electrodes, and the online reference channel Cpz was added back to the data. After filtering the EEG data from 1 to 48 Hz with a 5000-order finite impulse response (FIR) filter, it was segmented into 4-s epochs. Based on independent component analysis (ICA) performed in EEGLAB, we visually identified components related to eye blink artifacts (in general, 1–3 components) and removed them (Jung et al., 1997; Vigário, 1997; Delorme and Makeig, 2004). Noisy epochs were automatically removed using a simple threshold filter (−95 μV, +95 μV) on the segmented data.

#### Source reconstruction

To investigate brain activity in the motor network, we mapped the scalp EEG recordings to the cortical surface using inverse modeling.

We used the Freesurfer average brain (fsAverage; FreeSurfer Wiki) as a template for the source analysis. The electrode location and the template were used to set up a boundary-element method (BEM), representing three shells: brain, skull, and scalp. Within each shell, electric conductivity is assumed to be homogeneous. We used the fsAverage standard head and the standard electrode locations accompanied with MNE Python (Gramfort et al., 2010; Gramfort et al., 2013; Gramfort et al., 2014). We computed the forward model with perpendicular dipole orientations (Nunez et al., 1997; Srinivasan et al., 2006). The resulting two-dimensional lead field matrix with a dimension of 62 electrodes by 2052 voxels was exported to MATLAB for further analysis.

For computing the inverse solution, we used exact low-resolution brain electromagnetic tomography (eLORETA; Pascual-Marqui et al., 2011) implemented in the MEG/EEG toolbox of Hamburg (METH,^1^ developed by Guido Nolte).

#### Brain atlas and parcellation

As we were primarily interested in modeling connectivity changes between M1 and dPMC, we used the Human Connectome Project (HCP) Atlas-based parcellation template (Glasser et al., 2016) to define the voxels corresponding to the brain areas. In the HCP Atlas, M1 is defined as a single region of interest (ROI), whereas the premotor cortex (PMC) consists of multiple subregions. Regarding the dPMC, we considered only two ROIs, areas 6a and 6d, but not the premotor eye field (PEF). The time series from voxels within each ROI was aggregated into a signal by using singular value decomposition (SVD), keeping the first component, which explains the maximum variance. Note that we applied SVD on the band-pass filtered signals of each ROI in the beta frequency band (13–30 Hz), where a fourth-order Butterworth filter was used forward-backward (to compensate for the phase shift) for band-pass filtering the signals.

#### Power and connectivity analysis

The beta frequency power of each ROI was computed from the mean square of the beta frequency band signal of each ROI.

For quantifying functional connectivity between two ROIs, we computed the imaginary part of coherency (imCoh; Nolte et al., 2004) and the phase slope index (PSI; Nolte et al., 2008) in the beta frequency band (13–30 Hz). Using a customized MATLAB script, we computed the complex coherence of two narrow-band signals of the i-th and j-th ROIs as follows (Equation 1) (Idaji et al., 2022):


$${\mathrm{Coh}}_{\mathrm{ij}}=\frac{{\langle X_{i}(t)X_{j}^{*}(t)\rangle }_{t}}{\sqrt{{\langle {|X_{i}(t)|}^{2}\rangle }_{t}{\langle {|X_{j}(t)|}^{2}\rangle }_{t}}}$$ (1)

where $X_{i}(t)$ is the analytic signal of the i-th ROI computed from the Hilbert transform of the narrow-band signal (here in the beta frequency band), and ${\langle .\rangle }_{t}$ is the mean operator over time. For quantifying phase synchronization, we used the absolute value of the imaginary part of the coherency in Equation 1. Coherence (i.e., the absolute value of coherence) quantifies the strength of phase synchronization between two time series at a specific frequency. However, coherence is not robust to volume conduction. Therefore, the imCoh is used as a measure of phase synchronization, which is robust to volume conduction. The absolute value of imCoh reflects the strength of functional connectivity, which is only non-zero for non-zero lag synchronization.

PSI was used to quantify directed connectivity. We used the original implementation of PSI (Nolte et al., 2008) provided in the METH toolbox. PSI’s non-normalized value can be computed from the following (Equation 2):


ψ˜ij=Im(∑f∈FCij∗(f)Cij(f+δf)) (2)

where $C_{\mathrm{ij}}(f)$ is the complex coherency between ROI i and j, Im(.) is the operator for taking the imaginary part of a complex number, F is the set of all the frequency bins of interest, δf is the frequency resolution, and the ${\left(.\right)}^{*}$ operator is the complex conjugate.

In METH’s implementation, data is first epoched (we used a 4-s epoch length). Afterwards, for each epoch, the cross-spectra between and within ROIs are computed using fast Fourier transform (FFT; we set the frequency resolution of FFT to 0.5 Hz with a 1 s overlapping window). The function then computes the complex coherency of the i-th and j-th ROIs by averaging the normalized cross-spectrum between the two ROIs over the epochs at each frequency f . Note that the complex coherency computed from Equation 1 is equivalent to the average of the complex coherency as computed by METH’s PSI function over frequency bins of interest (Bruns, 2004).


$$\psi =\tilde{\psi }/\mathrm{std}(\tilde{\psi })$$ (3)

Then, as it is convenient, ${\tilde{\psi }}_{\mathrm{ij}}$ was normalized by an estimate of its standard deviation (Equation 3), which has been estimated by the jackknife method, resulting in a corresponding pseudo z-score (Nolte et al., 2008; Nolte et al., 2010). In general, absolute values greater than 2 are considered significant.

PSI measures the direction of information flow by establishing the driver-recipient relationship between the signals originating from the corresponding ROIs. Larger PSI values along with its pseudo z-score indicate a stronger deviation from 0 (where a PSI value of zero indicates no directionality) suggesting that a directed information transfer exists. Note that the magnitude of PSI does not reflect the interaction strength (Nolte et al., 2008). A positive sign of $\;\psi_{\mathrm{ij}}$ indicates that the signal from the i-th ROI leads (driver) the signal in the j-th ROI (receiver). Note that $\;\psi_{\mathrm{ij}}=-\psi_{\mathrm{ji}}$ .

In our study, positive values of PSI indicate a signal from M1 leading the signal in area 6a or 6d; assuming that M1 is the driver (i-th ROI) and area 6a or 6d (j-th ROI) is the receiver. For bidirectional (or unknown) coupling, a finding that, e.g., i-th ROI drives j-th ROI does not imply that j-th ROI has no impact on i-th ROI. Rather, one cannot make a statement about the reverse direction.

These signed PSI values thus allow for an interpretation of the directionality of neural interactions. However, at the group level, it is crucial to consider both the direction (sign of PSI) and the presence of a unidirectional information flow. Since averaging signed PSI values can lead to information loss due to potential cancelation effects, we additionally analyzed the absolute PSI values (absPSI). The absPSI provides a direction-independent measure of directed connectivity and reflects the overall presence of unidirectional information flow between the ROIs. A reduction in absPSI indicates weaker evidence for directional interactions. By analyzing both aspects—signed PSI for directionality and absPSI for magnitude—this dual approach enables a more comprehensive and robust interpretation of the underlying connectivity patterns.

## Results

We included 22 young, healthy participants (23.1 ± 2.9 years, mean age ± SD, range 18–32; 10 male). Two (out of 24) participants had to be excluded due to technical issues. We found no significant differences in terms of duration of night-sleep (sham: 6.8 ± 1.2 h; real: 6.7 ± 1.0 h; *p* = 0.599) and alertness/vigilance before the training and retest sessions as assessed with the Stanford-Sleepiness-Scale (sham: 1.8 ± 0.6; real: 1.9 ± 0.5; *p* = 0.083) and before retest (sham: 1.4 ± 0.6; real: 1.5 ± 0.6; *p* = 0.428).

Apart from a tingling skin sensation or flickering visual sensations when ramping up the current, no side effects of the stimulation were reported. Efficient blinding of participants with respect to real or sham stimulation was confirmed by the fact that the impression of real or sham stimulation matched the actual type of tDCS in only 27% of experiments (chi-squared test: *p* = 0.746). Real tDCS was correctly identified in only 7 of 22 cases. 4 of the 22 participants correctly identified the type of tDCS in both experimental sessions.

### Training-induced online motor performance gains

RmANOVA was used to conduct the PI across the training session with post-training *Intervention* (sham and real tDCS) and *Block* (B1, …, B14) as within-subject factors revealed a significant main effect of *Block* [*F*(4.279,89.874) = 32.245, *p* < 0.001]. However, there was no significant effect of *Intervention* [*F*(1,21) = 2.462, *p* = 0.132], or a significant interaction of the two factors [*F*(5.777,121.308) = 1.282, *p* = 0.272]. The mean PI at the beginning of training (first block) amounted to 20.5 ± 8.3 for the post-training sham intervention and 22.9 ± 7.4 for the real tDCS intervention and did not significantly differ between interventions (*p* = 0.073). Performance improved across training and reached similar PI values at the end of the training session (EoT, sham: 33.2 ± 8.9; real: 33.0 ± 8.2; *p* = 0.794).

**Figure 2:**
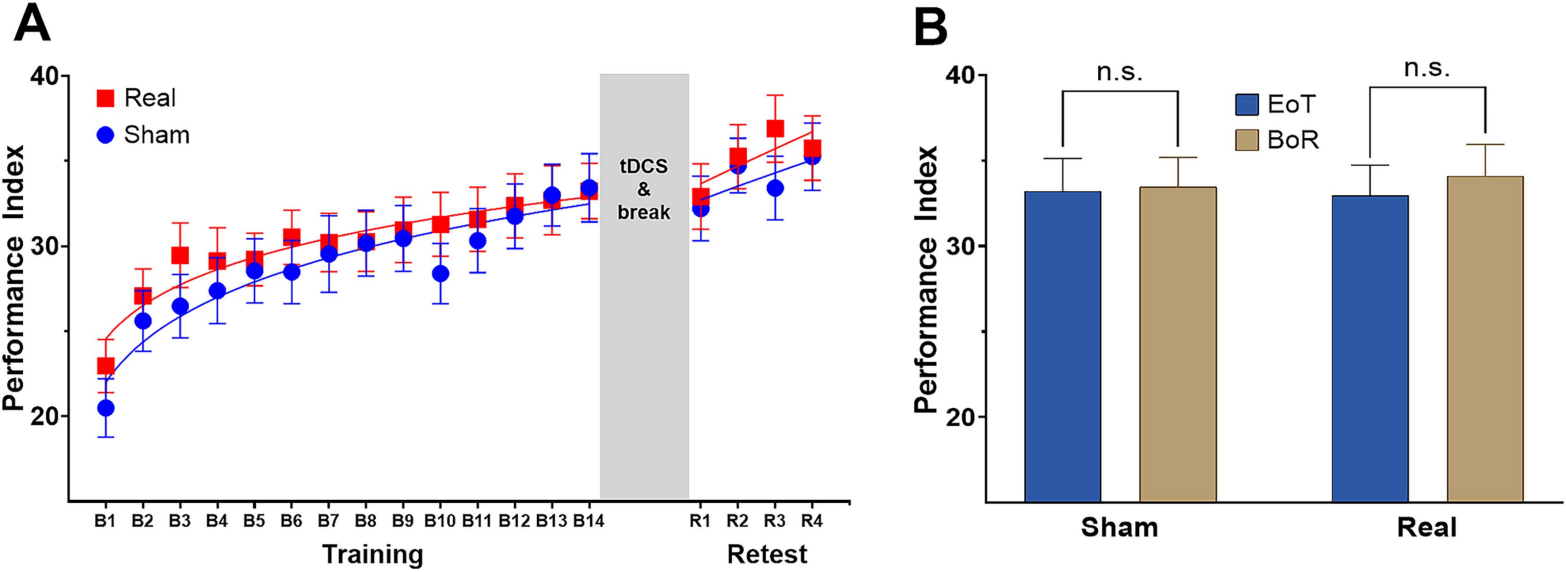
Behavioral results. **(A)** Motor performance. Mean Performance Index within a given block for the sham tDCS (Sham) and real tDCS (Real) experiment across blocks of task execution during Training (blocks B1–B14) and Retest (blocks R1–R4). **(B)** Mean Performance Index difference at end of training (EoT, mean PI across the last 2 blocks of the training session) and beginning of the delayed retest (BoR, mean PI across the first 2 blocks of delayed retest). Error bars represent standard error of the mean. tDCS, transcranial direct current stimulation. n.s., not significant.

These results indicate that participants improved task performance across the training session significantly and comparably in both experiments (Figure 2A). Therefore, EoT performance was used as the baseline against which the magnitude of between-session offline performance changes was assessed.

### No evidence of post-training tDCS effects on motor memory consolidation

To examine whether post-training stimulation (sham and real tDCS) affected task performance during the delayed retest, we conducted a rmANOVA on the PI values across the delayed retest blocks. This analysis revealed a significant main effect of *Block* [*F*(3,63) = 79.591, *p* < 0.001], but no significant effect of *Intervention* [*F*(1,21) = 74.678, *p* = 0.25] nor a significant interaction of the two factors [F(3,63) = 23.309, *p* = 0.082]. These results provide no evidence that immediate post-training tDCS influenced online learning dynamics or overall task performance during delayed retesting.

Between-session offline performance changes (ΔPI_offline_) were quantified by comparing PI at the EoT baseline with PI at the BoR (mean of the first two retest blocks). ΔPI_offline_ served as an index of the effectiveness of offline motor memory consolidation processes. Across both experiments, mean PI increased incrementally from 33.1 ± 8.4 at EoT to 33.8 ± 8.1 at BoR. Following sham stimulation, the mean PI at BoR was 33.5 ± 7.9, compared with 34.1 ± 8.5 after real stimulation (Figure 2B). This corresponded to a numerically larger increase of the PI between BoR and EoT after real post-training tDCS (1.12 ± 3.55) vs. after sham tDCS (0.25 ± 4.43). However, rmANOVA with the factors *Intervention* (sham, real tDCS) and *Session* (EoT, BoR) showed neither significant effects of *Intervention* [*F*(1,21) = 0.747, *p* = 0.816], of *Session* [F(1,21) = 1.197, *p* = 0.286], nor a significant interaction of both factors [F(1,21) = 0.596; *p* = 0.449]. Overall, these results provide no evidence of a meaningful post-training tDCS effect on subsequent offline motor memory consolidation at the behavioral level (Figure 2B).

### No evidence of tDCS effects on regional spectral power in beta frequency in the left primary motor cortex and left dorsal premotor cortex

**Figure 3:**
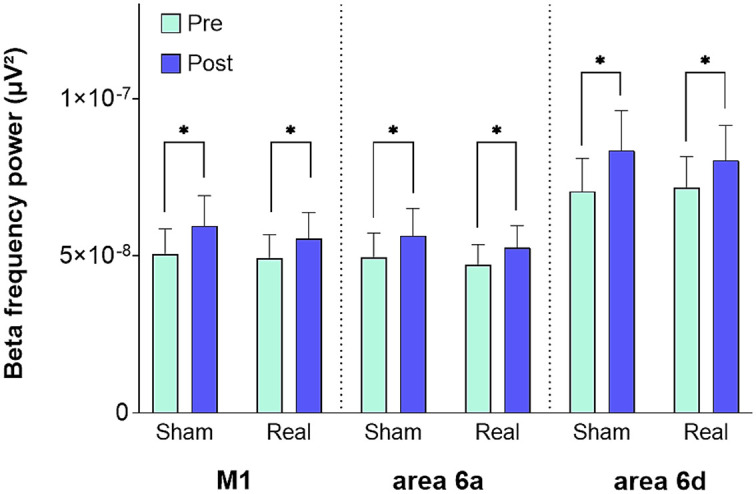
Beta frequency power in M1 and dPMC (areas 6a and 6d). Mean beta frequency power in each region of interest (M1, area 6a, and 6d) before (Pre) and after (Post) tDCS in sham (Sham) and real (Real) tDCS session. Error bars represent standard error of the mean. *p < 0.001. M1, primary motor cortex; area 6a/area 6d, subareas of the dorsal premotor cortex; tDCS, transcranial direct current stimulation.

We computed EEG beta frequency power (13–30 Hz) before training (pre EEG) and after post-training tDCS (post EEG) to investigate whether and how training and tDCS (real and sham) affected beta frequency power in M1 and left dPMC with areas 6a and 6d. Across both experiments, we observed a significant beta frequency power increase in all 3 regions of interest (M1, area 6a, area 6d) following post-training tDCS [M1, factor *Time* (pre EEG, post EEG), F(1,21) = 35.81; *p* < 0.001; area 6a, *Time* F(1,21) = 27.701; *p* < 0.001; area 6d, *Time* F(1,21) = 24.577; *p* < 0.001; Figure 3]. However, there was no significant interaction of *Time x Intervention* in any of these regions (all *p* ≥ 0.48) that would suggest significant tDCS-induced effects on beta frequency oscillatory activity.

### No evidence of tDCS effects on interregional coherence between the left primary motor cortex and left dorsal premotor cortex

**Figure 4:**
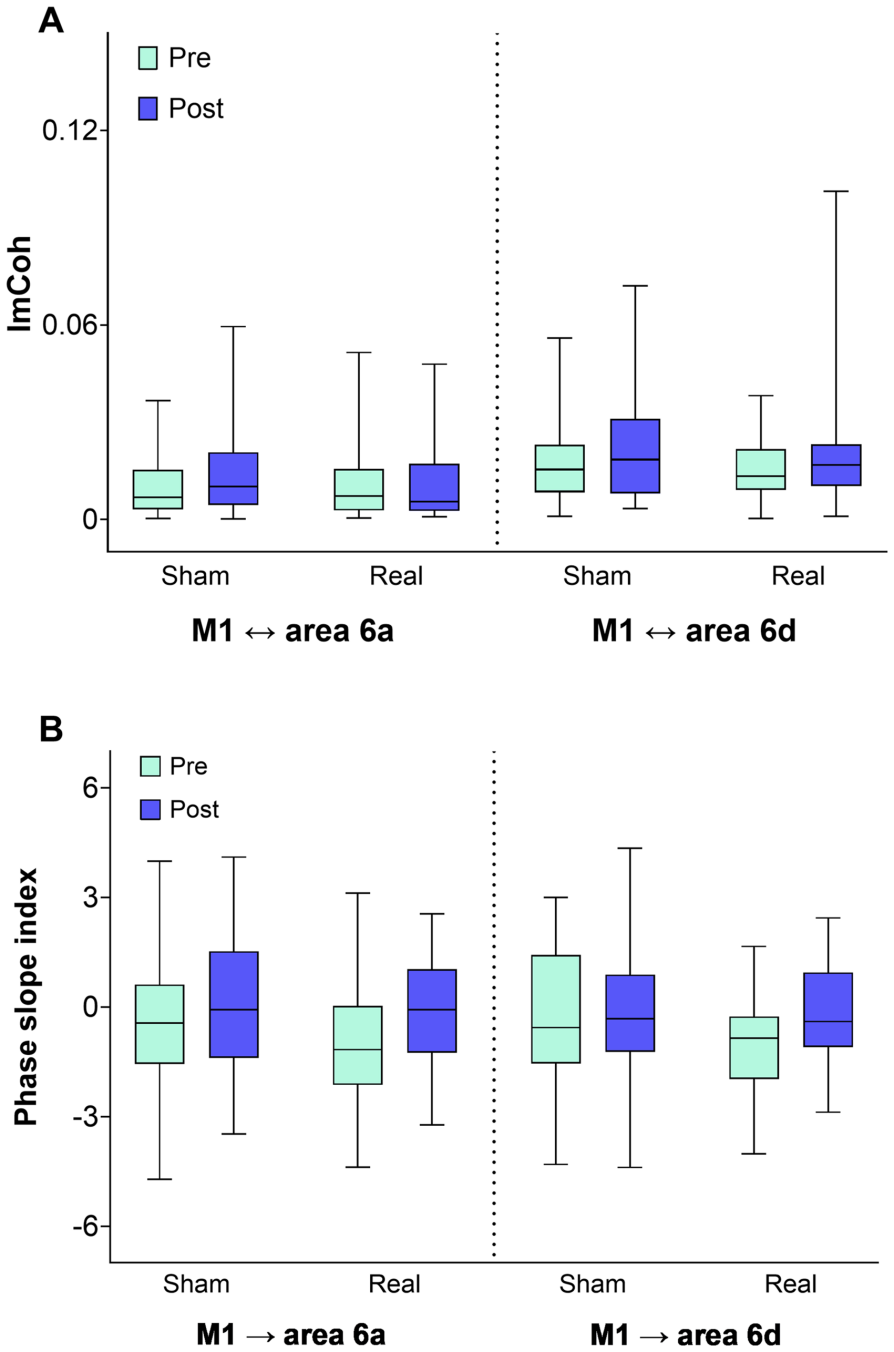
Connectivity results. **(A)** Interregional coherence between left M1 and left dPMC (areas 6a, 6d). Box plots of the imaginary part of coherency (imCoh) in beta frequency band between the regions M1 and areas 6a (M1↔6a) and 6d (M1↔6d) of dPMC before (Pre) and after (Post) tDCS in sham (Sham) and real (Real) session. **(B)** Phase slope index. Box plots (min to max) of the phase slope index between M1 and areas 6a (M1 → 6a) and 6d (M1 → 6d) before (Pre) and after (Post) tDCS in sham (Sham) and real (Real) session. Positive values designate predominant information flow from M1 to area 6a/area 6d, respectively. Error bars represent standard error of the mean. No significant changes were observed. M1, primary motor cortex; dPMC, dorsal premotor cortex; area 6a/area 6d, subregions of dPMC; tDCS, transcranial direct current stimulation.

We then examined whether the imaginary part of coherency between left M1 and left dPMC was influenced by post-training tDCS. For both prespecified connections (M1 ↔ area 6a; M1 ↔ area 6d), rmANOVA revealed no significant main effect of the factors *Time* [pre EEG and post EEG; M1 ↔ 6a: *F*(1,21) = 1.751, *p* = 0.200; M1 ↔ 6d: F(1,21) = 3.403, *p* = 0.079], *Intervention* [sham and real tDCS; M1 ↔ 6a: F(1,21) = 0.020, *p* = 0.889; M1 ↔ 6d: F(1,21) = 0.428, *p* = 0.520], and no significant interaction of both factors [*Time x Intervention* M1 ↔ 6a: F(1,21) = 1.483, *p* = 0.237; M1 ↔ 6d: F(1,21) = 0.368, *p* = 0.550]. These observations provide no evidence of a meaningful tDCS-induced modulation of post-stimulation functional connectivity between left primary and left dPMC as indexed by the imCoh in the beta frequency band (Figure 4A).

### No evidence of meaningful tDCS effects on phase slope index between the left primary motor cortex and the left dorsal premotor cortex

We further explored whether PSI in the beta frequency band, reflecting the directed information flow between M1 and areas 6a and 6d, was modulated by the type of post-training stimulation. The direction of information flow is indexed by the sign of PSI. Figure 4B illustrates that pre-training PSI values were on average negative. Because the PSI was defined to designate predominant information flow from M1 to area 6a/area 6d (see Methods), more negative values indicated predominance of directional interaction from dPMC to M1. Following training, on average, values tended toward 0 or became positive, suggesting reduced evidence of directional interaction from dPMC to M1, or even reversal to predominant interaction from M1 to dPMC (Figure 4B).

RmANOVA on the original (signed) values of PSI related to directed information flow between M1 and area 6a (M1 → 6a) revealed a significant effect of *Time* [*F* (1,21) = 6.303, *p* = 0.020]. However, there was neither a significant effect of *Intervention* [F (1,21) = 0.55, *p* = 0.467] nor a significant interaction of *Intervention x Time* [F(1,21) = 0.802, *p* = 0.381], suggesting that motor training, but not tDCS, had modulated M1 → 6a information flow. RmANOVA on the PSI related to information flow from M1 to area 6d (M1 → 6d) revealed a weak trend for the main effect of *Time* (*F* = 3.154, *p* = 0.090) and a trend toward a significant *Intervention x Time* interaction (*F* = 4.076, *p* = 0.056). This interaction was driven by an increase in the original (signed) PSI following real tDCS, indicating that the flow of information from M1 to area 6d became relatively stronger.

To investigate the possibility that more positive PSI values after training and stimulation had arisen by cancelation effects from averaging PSI values of opposite signs, we additionally analyzed the absolute PSI values (absPSI). The absPSI is independent of the direction of information flow and, therefore, reflects the overall presence of unidirectional information flow between the ROIs. Reduction in the mean of absPSI indicates weaker evidence for any directional interaction. RmANOVA applied to absPSI revealed no significant main effect of *Intervention* (M1 ↔ 6a, *p* = 0.902; M1 ↔ 6d, *p* = 0.182), and *Time* (M1 ↔ 6a, *p* = 0.586; M1 ↔ 6d, *p* = 0.255), nor for the interaction of both factors (*Intervention x Time* M1 ↔ 6a, *p* = 0.220; M1 ↔ 6d, *p* = 0.996). Taken together, the findings show no meaningful evidence that motor sequence training or the post-training real tDCS intervention altered directed interactions between M1 and dPMC areas 6a or 6d (see Supplementary Figure 1).

As indicated above, we found a trend for a stimulation effect on the training-dependent increase of information flow from M1 to area 6d (M1 → 6d) as evidenced by a shift from negative PSI values to more positive ones. To explore this association further, we specifically investigated changes in absPSI in this condition. AbsPSI in real tDCS decreased from pre-stimulation (abs[mean(PSI)] = 0.983) to post-stimulation (abs[mean(PSI)] = 0.157; T = –3.223, *p* = 0.004, df = 21), whereas absPSI in sham tDCS remained stable (pre-stimulation abs[mean(PSI)] = 0.31, post-stimulation abs[mean(PSI)] = 0.27; T = –0.113, *p* = 0.911, df = 21). This indicated that the trend for a relative increase of signed PSI (M1 → 6d) after real tDCS was more likely related to a homogeneous decrease of directed information flow between area 6d and M1 (corresponding to a relative increase from M1 to area 6d) rather than to the reversal of the information flow in some subjects and unchanged information flow in others.

### Relationship of post-training motor memory consolidation and EEG metrics

Since post-training tDCS had no meaningful effect on motor memory consolidation, as measured by between-session performance changes, we subsequently sought to obtain insight into neurophysiological mechanisms of motor memory consolidation by combining experiments with real and sham stimulation, exploiting the interindividual variance of ΔPI_offline_ (between-session changes of the PI from EoT to retest BoR, see Methods). In this exploratory analysis, we investigated the relationship between ΔPI_offline_ and (a) measures of regional oscillatory brain activity (beta frequency power), (b) functional connectivity (imCoh), and (c) directed information flow (PSI) measures between M1 and dPMC (areas 6a and 6d). As we were interested in the post-stimulation status of the ROIs and interactions between the ROIs, we correlated the EEG metrics derived from post-stimulation EEG recordings after both interventions (sham and real tDCS) with ΔPI_offline_.

**Figure 5:**
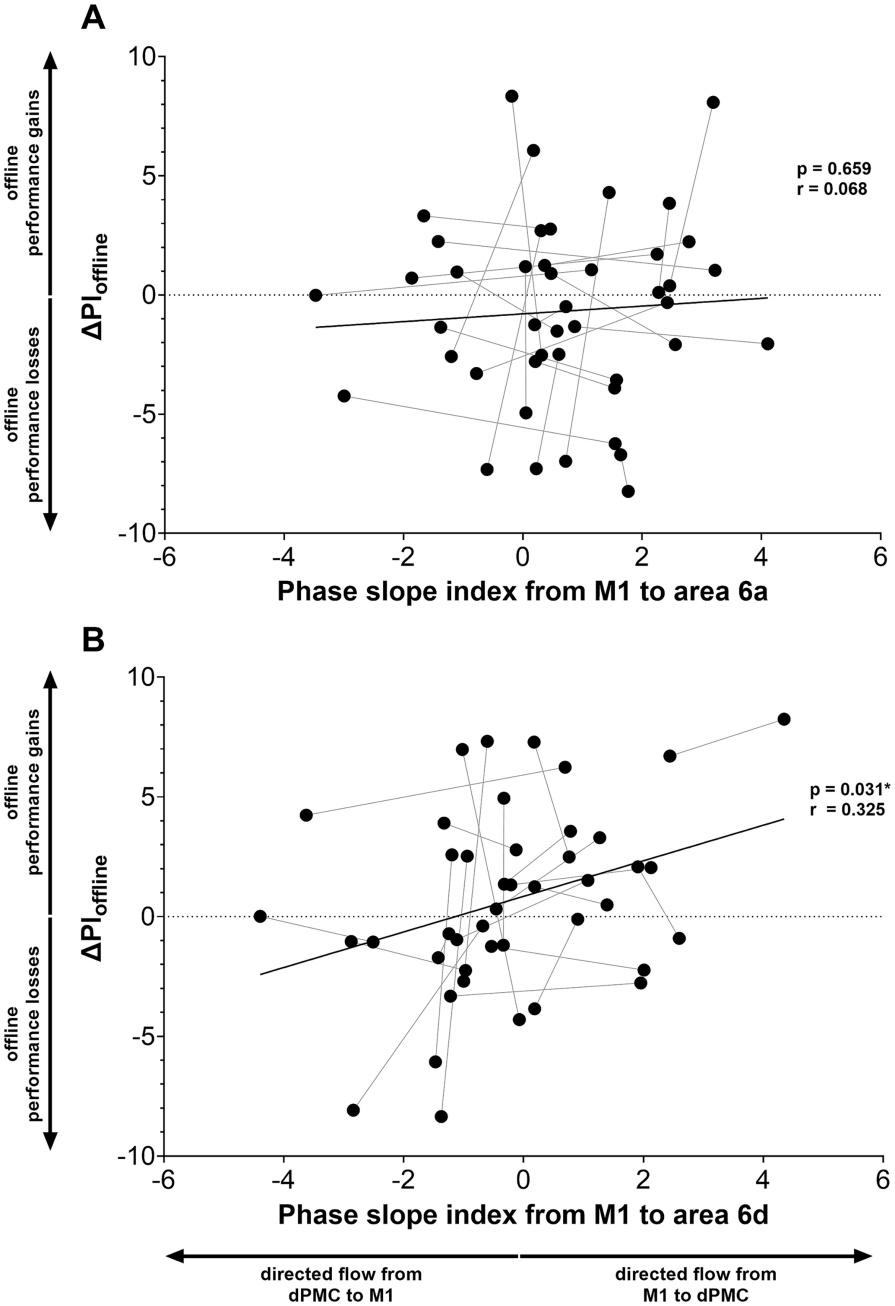
Relationship of between-session performance changes and phase slope index between M1 and areas 6a and 6d of dPMC. Offline Performance Index change (ΔPI_offline_, *y*-axis), calculated as the difference between performance at the start of the retest and at the end of training. Positive values indicate between-session (offline) increases in PI, while negative values indicate decreases. The *x*-axis shows the phase slope index between M1 and area 6a **(A)** or area 6d **(B)** of the dPMC after post-training tDCS. Exploratory analysis of data from 22 participants, each completing two sessions (44 data points); participant-level pairing is indicated by gray lines connecting the two observations for each of the 22 participants. Pearson’s *r* and *p*-values displayed for each analysis. M1, primary motor cortex; dPMC, dorsal premotor cortex; area 6a/6d, subregion of the dorsal premotor cortex. PI = Performance Index. *uncorrected *p* < 0.05.

In this exploratory analysis, combining data from real and sham tDCS experiments, we did not find significant correlations between post-stimulation beta frequency power in all ROIs (all *p* ≥ 0.887) or imCoh (all *p* ≥ 0.496) and ΔPI_offline_ (see Supplementary Table 1). Likewise, there was no significant correlation between the post-stimulation PSI values of the connection between M1 and area 6a (M1 → 6a) and ΔPI_offline_ (*p* = 0.659; Figure 5A). However, we observed a significant correlation between the post-stimulation signed PSI values of the connection of M1 with area 6d (M1 → 6d) and ΔPI_offline_ (uncorrected *p* = 0.031). The Pearson correlation coefficient was 0.325, suggesting a positive association. This may suggest that stronger directional information flow from M1 to area 6d of the dPMC could be associated with improved post-training offline motor memory consolidation (Figure 5B). However, this exploratory observation should be interpreted with caution, as it did not remain significant after correction for multiple comparisons.

## Discussion

The present study used behavioral testing, tDCS of M1, and high-density EEG to study the malleability of the post-training motor memory consolidation process by offline non-invasive brain stimulation and gain novel insight into its mechanisms. However, there was no statistical evidence of an advantage of post-training real tDCS over sham stimulation in the magnitude of between-session offline task performance changes, despite a small numerical difference. Consequently, our study failed to provide evidence that post-training tDCS modulates the offline motor memory consolidation process in young, healthy adults. While some studies have suggested that tDCS applied after motor training can enhance offline motor memory consolidation in both young and older adults (Tecchio et al., 2010; Krause et al., 2016; Rumpf et al., 2017), others have found no such effect when tDCS was administered after training (Reis et al., 2015; Chen et al., 2020). These latter studies instead propose that the beneficial effects of tDCS on motor memory consolidation may require an online interaction between tDCS and ongoing practice (Reis et al., 2009; Saucedo Marquez et al., 2013; Reis et al., 2015; Kim et al., 2021). Alternatively, given the young age of participants in the present study and differences in the applied tasks, it is possible that performance improvements during the initial training session approached a ceiling level, leaving limited room for offline between-session performance gains, including those potentially induced by tDCS.

Overall, our findings contribute to the growing body of evidence emphasizing the variability of tDCS-induced effects across various motor and cognitive tasks (Saucedo Marquez et al., 2013; Puri et al., 2021; Hsu et al., 2025). Although tDCS is a relatively simple stimulation technique in terms of its hardware, increasing evidence suggests that variability in protocol parameters—such as electrode montage, stimulation intensity, stimulation duration, and task complexity, along with inter-individual differences in responsiveness to tDCS, may contribute to the inconsistency of outcomes (Buch et al., 2017).

### No evidence of meaningful post-training tDCS effects on oscillatory brain activity, functional connectivity, and directed information flow

Beta frequency power was enhanced after post-training tDCS, but this change was not significantly different between the real or sham tDCS intervention. Some studies (Song et al., 2014; Masina et al., 2021; Xiao et al., 2023; Bernardes et al., 2024) have reported that tDCS applied to motor cortical areas alters beta-frequency power. For instance, it has been demonstrated that specifically beta frequency power—detected in a single channel EEG with the electrode placed between Fp1 and Fp2—increased promptly after starting tDCS with anodal stimulation over the left dorsolateral prefrontal cortex, while other frequency bands remained unaffected (Song et al., 2014). Other studies (Stillman et al., 2013; Kim et al., 2020; Ghafoor et al., 2022; Liu et al., 2023; Vimolratana et al., 2024) reported no significant effects of tDCS on beta frequency power. In stroke patients, no significant beta frequency power modulation was observed in resting-state EEG following 5 days of real tDCS combined with physical therapy, compared to sham intervention (Vimolratana et al., 2024). Another study in stroke patients also observed no significant beta frequency power changes after real and sham tDCS (Liu et al., 2023). Our findings indicate that training-induced increases in beta frequency power were independent of the tDCS intervention, and, hence, were likely induced by prior motor training.

Functional connectivity analysis, as assessed by imCoh between M1 and dPMC, revealed no significant alterations through post-training tDCS. However, average values were numerically slightly higher after real tDCS compared to sham stimulation. So far, only a few studies have specifically examined how tDCS affects functional connectivity using imCoh. This is noteworthy, as imCoh has been shown to represent a particularly reliable metric for assessing true brain connectivity—it reduces zero-lag correlations and helps minimize artifacts from volume conduction. Absence of evidence of a tDCS effect on imCoh between M1 and dPMC is in line with results from Schoellmann et al. (2019), who investigated tDCS effects on cortico-cortical coherence in the beta frequency band in patients with Parkinson’s disease and healthy controls performing an isometric precision grip task. In contrast, a study by Notturno et al. (2014) reported that anodal tDCS over the left M1 led to a significant increase in beta-band imCoh at rest—particularly between M1 and bilateral parietal as well as right frontal regions. These latter results suggest that tDCS may influence not only local activity but also more widespread connectivity within the motor network. Compared to their broader network-level analysis, our focus on a specific connection between two anatomically close regions may have been less sensitive to detecting such widespread changes.

In general, pre-training PSI values indicated predominance of directional interaction from dPMC toward M1. Conversely, post-intervention values trended toward less negative or more positive values, suggesting decreased evidence of directional interaction from dPMC toward M1 or, conversely, a greater influence of M1 on dPMC. There were subtle differences between M1 → area 6a and M1 → area 6d information flow. Following the intervention, directed information flow (PSI) pertaining to the information flow from M1 to area 6a (M1 → 6a) indicated a reduction in directed information flow independent of stimulation type. Regarding information flow for the connection M1 → 6d, we found a trend toward an interaction between Time and Intervention. For real tDCS only, baseline predominant information flow from area 6d to M1 lost its predominant direction, favoring relative strengthening of M1 to area 6d information flow after the intervention. A decrease in mean PSI magnitudes (absPSI) after real tDCS suggested a homogeneous response across participants. Although this result raised the possibility of a region- and stimulation-specific effect, its significance remains uncertain due to the exploratory nature of the analysis. Our literature review identified no studies investigating the effects of tDCS on PSI.

### Associations of training-induced local power changes and M1–dPMC interaction with motor memory consolidation

We found an enhancement in beta frequency power after initial motor sequence training that appeared to be independent of tDCS. Several studies have observed beta frequency power changes in motor cortices after motor training (Mary et al., 2015; Moisello et al., 2015; Nelson et al., 2017; Espenhahn et al., 2019; Dyck and Klaes, 2024). Most findings of post-training increase in beta frequency power have been interpreted to reflect increased cortical excitability in motor cortical areas promoting plastic changes (Meissner et al., 2018; Dyck and Klaes, 2024) with no direct relationship to training-induced performance gains or even later performance gains corresponding to consolidation. Espenhahn and co-workers trained healthy adults on a novel wrist flexion/extension tracking task (Espenhahn et al., 2019). Their results showed no correlation between resting beta frequency power and late motor performance 24 h after training, despite evidence of an immediate post-training increase in beta frequency power. The authors interpreted this increase as a plasticity-related modulation of beta activity. Beta power increase—particularly in the resting state after intensive practice—has even been suggested by some authors to reflect more than the initial plastic processes of motor learning and plasticity-related activation, but as candidate markers of plasticity engagement and may be associated with subsequent motor consolidation and optimization. However, this remains a hypothesis (Ghilardi et al., 2021). Boonstra et al. (2007) reported an inverse relationship between beta frequency power in M1 and the force exerted during a complex bimanual motor task. Although these EEG findings indicated that a link between early post-training motor cortex activity and the behavioral adaptations occurring during the learning phase may exist, the results are not comparable with the present study, as EEG was recorded during movement in Boostra and co-workers’ study (Boonstra et al., 2007), but at rest in our study. Because in the present study motor performance did not improve in the retest compared to the level attained at the end of training, we assume that post-training changes of oscillatory activity likely reflect a short-lived expression of cortical plasticity (Moisello et al., 2015; Espenhahn et al., 2019; Tatti et al., 2020) with no direct mechanistic connection to motor consolidation. In summary, although our findings replicate prior evidence that motor sequence training increases beta-frequency power in motor cortical areas (M1 and dPMC), we found no evidence that these training-related increases in beta oscillations are associated with the outcome of subsequent offline motor memory consolidation at the behavioral level.

Moreover, functional connectivity as measured by imCoh showed no relevant association with between-session task performance changes. To our knowledge, no previous studies have specifically examined the relationship between imCoh and motor learning or motor memory consolidation. However, other functional connectivity measures have produced mixed findings. For instance, functional connectivity between M1 and parietal regions has been linked both positively (Mary et al., 2017) and negatively (Wu et al., 2014) to motor learning outcomes. Wu et al. (2014) found that resting-state beta-band coherence before training was predictive of motor learning across sessions. Their analysis revealed significant clusters, including over left M1 (e.g., electrode C3), suggesting that stronger resting-state connectivity may support offline motor memory consolidation processes occurring between training and retest. Similarly, Sugata et al. (2020) reported that resting-state (seed-based) beta-band connectivity across multiple networks was associated with motor performance gains at delayed retesting. In their study, the strongest (negative) correlation was observed in the left superior temporal gyrus, indicating that subjects with strong resting-state functional connectivity in the beta band between the left M1 and the left superior temporal gyrus had low performance in the subsequent motor learning task (Sugata et al., 2020). While these studies suggest that functional connectivity may be linked to immediate performance gains and learning, none have explicitly examined post-training motor memory consolidation. As such, they do not directly inform our finding of no observed association between imCoh and the post-training offline motor memory consolidation process.

In an exploratory analysis, PSI (M1 → 6d) effective connectivity revealed a significant (uncorrected for multiple comparisons) correlation with between-session task performance changes. This may suggest that stronger directed interactions from M1 to area 6d (or attenuation of directed interaction from area 6d to M1) may be associated with enhanced motor memory consolidation. Although this potential association may suggest an underlying neural mechanism for motor memory consolidation, it should be interpreted with caution due to the exploratory nature of the analysis. If this interpretation is accepted, alterations in information flow between M1 and area 6d could indicate a possible competitive interaction within the motor consolidation network between M1 and dPMC, compatible with findings by Veldman et al. (2018), who reported that changes in PSI between M1 and dPMC were associated with different stages of motor learning. Notably, during the consolidation phase, they observed that directed information flow from sensory and parietal areas toward M1 correlated with performance stabilization. Although their analysis focused on cortical connections (e.g., S1 → M1, parietal → M1) that differ from the M1–dPMC connection examined in the present study, their findings further support the notion that consolidation is not only dependent on local activity but also on coordinated interactions between distributed brain areas (Veldman et al., 2018). Along with these findings (Veldman et al., 2018), our results highlight the potential role of directional resting-state connectivity as a neural substrate of motor memory consolidation. However, it remains unclear whether directional influences reflect stable trait-like properties or adaptive processes induced by training. Further research is needed to clarify whether enhanced directional coupling from M1 to higher-order motor areas, such as PMC, facilitates memory consolidation through top-down motor control or reflects reorganization within the motor learning network.

This question becomes particularly relevant in light of converging evidence indicating the critical role of dPMC in both online and offline phases of motor memory formation. dPMC is known to inhibit M1 and influence M1 plasticity via interregional connections (Gerschlager et al., 2001; Münchau et al., 2002; Huang et al., 2009; Huang et al., 2018). Its involvement in motor consolidation has been demonstrated through various non-invasive brain stimulation studies. While some reported beneficial effects on consolidation excitatory stimulation of dPMC (Boyd and Linsdell, 2009), others found impaired retention or no effect depending on stimulation timing, protocol, and population (Kantak et al., 2012; Rumpf et al., 2017; Pollok et al., 2021). These divergent outcomes underscore the complexity of dPMC’s role within the motor learning network. Thus, although tDCS did not promote the post-training offline motor memory consolidation process in our study, the directional interaction patterns between M1 and dPMC reported in previous work suggest that network-specific mechanisms, rather than general increases in excitability, may be more critical for promoting motor consolidation. Our findings are in line with this hypothesis. Future studies should further explore whether enhancing M1 → dPMC communication through targeted neuromodulation or task design can improve consolidation outcomes.

### Limitations

Our study has several limitations. First, the absence of a meaningful behavioral tDCS effect on motor memory consolidation restricted our ability to investigate the underlying physiological mechanisms, limiting analyses to between-subject variability. Moreover, given the young age of the participants, it is possible that task performance approached a ceiling during the initial training session, leaving limited room for further offline gains, including those potentially induced by tDCS. Future studies should therefore employ more demanding or adaptive tasks to increase the likelihood of detecting meaningful differences in motor memory consolidation. Second, we used ring electrodes for stimulation to enable the simultaneous EEG montage, whereas previous tDCS studies typically employ larger square electrodes. The use of ring electrodes may have reduced effective current flow, thereby weakening target stimulation. Third, for source reconstruction of the ROIs (M1 and areas 6a and 6d), we relied on a standard head model rather than individualized models derived from each participant’s MRI and EEG localizer. Using individualized head models would have provided more precise and accurate source reconstruction and potentially improved the spatial specificity of the stimulation. Finally, the observed PSI–motor memory consolidation association resulted from exploratory uncorrected post-hoc analyses and should therefore be considered preliminary and interpreted with caution.

## Conclusion

Our study showed that motor sequence learning was accompanied by significant and sustained increases in beta-frequency power within both the M1 and the dPMC, underscoring the relevance of beta oscillations in motor learning. However, post-training tDCS applied to M1 did not produce behaviorally meaningful changes in beta power, nor did it affect the offline motor memory consolidation process as indexed by between-session task performance changes. The trend toward reduced directed connectivity from dPMC (area 6d) to M1 following post-training real tDCS, together with exploratory evidence that decreased—or reversed—information flow from dPMC to M1 was associated with enhanced motor memory consolidation, may be consistent with the idea that dPMC exerts an inhibitory influence on M1 during the offline motor memory consolidation process. However, this interpretation remains speculative and warrants further investigation.

## Data Availability

The raw data supporting the conclusions of this article will be made available by the authors, without undue reservation.

## Footnotes

1
https://www.uke.de/english/departments-institutes/institutes/neurophysiology-and-pathophysiology/research/research-groups/index.html
