# Supplementary material for: Absence of evidence for post-training tDCS effects on motor memory consolidation and premotor–primary motor cortex interaction: a resting-state EEG study
Source: Front Hum Neurosci. 2026 Feb 13;20:1698460. doi: 10.3389/fnhum.2026.1698460 (PMC12946050; doi:10.3389/fnhum.2026.1698460)
Supplement: Supplementary file 1 [file Data_Sheet_1.docx]

Supplementary Material

# Supplementary data

**Accuracy of motor performance**

A rmANOVA of the accuracy measures showed no significant effects for the factors *Intervention* (sham and real tDCS) and *Block* (B1 – B14; R1 - R4) or the interaction of both factors in the training session (*Intervention* F(1,21)=0.297, p=0.591; *Block* F(13,273)=1.585, p=0.089; *Intervention x Block* F(13,273)=1.019, p=0.433) and in the delayed retest session (*Intervention* F(1,21)=0.349, p=0.561; *Block* F(3,63)=1.28, p=0.289; *Intervention x Block* F(3,63)=1.162, p=0.331). Collectively, this indicates that accuracy of motor sequence performance was not modulated by repeated practice or stimulation at group level.

**Task performance speed**

Average baseline task performance speed (mean time of correct sequences per block) in the first two blocks of the training amounted to 1.429 s (CI 1.33–1.527; mean speed ± SD: sham 1.492 s ± 0.36, real: 1.393 ± 0.331) and did not significantly differ between both interventions sham and real tDCS (F(1,23)=4.026, p=0.057). A rmANOVA over the 14 training blocks revealed a significant main effect of *Block* (F(3.692, 84.91)=46.728, p<0.001), indicating a significant change in performance speed during the motor training. The task performance speed in end of training (EoT; mean of the last two training blocks) before sham tDCS intervention was 1.102 ± 0.271 s and before real tDCS intervention 1.109 ± 0.257 s. There was no significant main effect of *Intervention* (F(1,21)=0.094, p=0.762), indicating similar task performance speed at EoT before sham and real tDCS. Similar to the PI analysis, the conducted rmANOVA with the factors *Intervention* (sham and real) and *Session* (EoT and BoR) revealed no significant effect for *Intervention* (F(1,21)=0.107, p=0.747) or *Session* (F(1,21) = 0.196, p = 0.663). There was no interaction of both factors (F(1,21)=0.498, p=0.488).

# Supplementary tables and figures

| *EEG Parameter* |  | *Pearson's correlation coefficient (r)* | *Significance (p-value)* |
| --- | --- | --- | --- |
| Post Beta Frequency Power | Region M1 | 0.004 | 0.982 |
|  | Area 6a | 0.022 | 0.887 |
|  | Area 6d | 0.007 | 0.962 |
| Post ImCoh | M1↔6a | -0.105 | 0.496 |
|  | M1↔6d | -0.053 | 0.735 |
| Post PSI | M1→6a | -0.063 | 0.683 |
|  | M1→6d | 0.325 | 0.031* |

**Supplementary Table 1:** Analysis of correlation between post intervention EEG metrics and ΔPI_offline_ - as indexed by change of the PI at the EoT to the PI at retest - after combining data from real as well as sham tDCS experiments. M1= primary motor cortex; area 6a/area 6d= subareas of the dorsal premotor cortex; imCoh= imaginary part of coherency; PSI= Phase slope index. * = significant, p≤0.05.


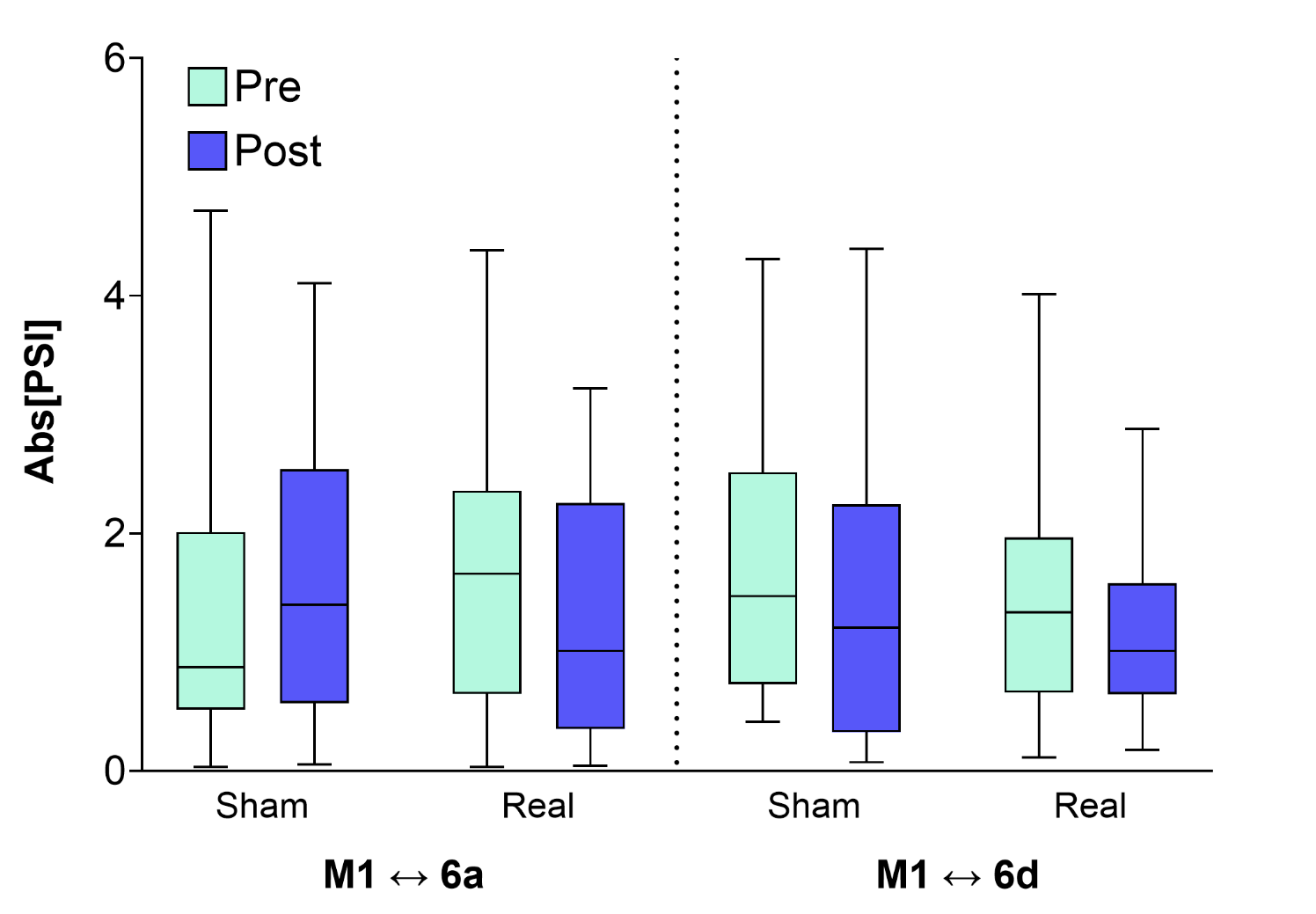


**Supplementary Figure 1: Absolute Phase slope index (absPSI).** Box plots (min to max) of the magnitude (absolute values) of phase slope index (abs[PSI]) between M1 and areas 6a (M1↔6a) and 6d (M1↔6d) before (Pre) and after (Post) tDCS in sham (Sham) and real (Real) session. M1= primary motor cortex; area 6a/area 6d= subregions of the dorsal premotor cortex; tDCS= transcranial direct current stimulation.
